# The Texas Pharmaceutical Association

**Published:** 1882-08-20

**Authors:** 


THE TEXAS PHARMACEUTICAL ASSOCIATION held its fourth annual meeting in Fort Worth, May 9th and 10th, the Second Vice-President, Leo Preuss, of Eunis, presiding. The Secretary read the annual address, containing valuable suggestions for the future welfare of the Association. Among the business transacted was the application for a charter, and the election of a Committee of Trustees. The following officers were elected to serve for one year: President, E. M. Wells, Fort Worth; Vice-Presidents—W. J. Morley, of Austin; T. W. Powell, of Fort Worth, and C. F. Hall, of Bryan; Treasurer, E. W. Lancaster, of Marshall; Secretary, W. H. Murdock, of Dallas. The next meeting will be held in Austin, the capital, on the second Tuesday of May, 1883. —*Exchange.*

